# Spatial Dependence of Local Density of States in Semiconductor-Superconductor Hybrids

**DOI:** 10.1021/acs.nanolett.4c03108

**Published:** 2024-10-18

**Authors:** Qingzhen Wang, Yining Zhang, Saurabh Karwal, Srijit Goswami

**Affiliations:** † QuTech and Kavli Institute of Nanoscience, Delft University of Technology, Delft 2600 GA, The Netherlands; ‡ QuTech and Netherlands Organization for Applied Scientific Research (TNO), Delft 2628 CK, The Netherlands

**Keywords:** Two-dimensional Electron Gas, Proximity Effect, Tunneling spectroscopy, Topological Superconductivity

## Abstract

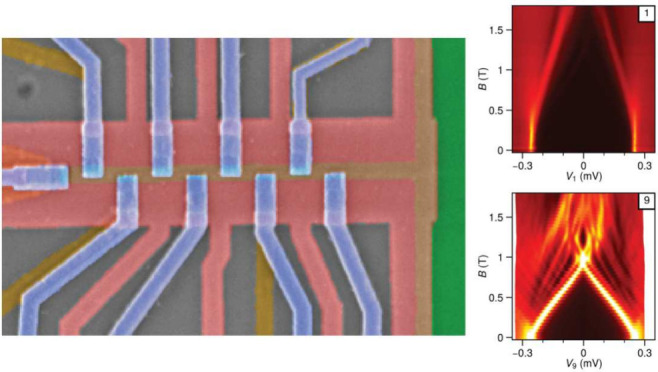

Majorana bound states are expected to appear in one-dimensional semiconductor-superconductor hybrid systems, provided they are homogeneous enough to host a global topological phase. In order to experimentally investigate the uniformity of the system, we study the spatial dependence of the local density of states in multiprobe devices where several local tunneling probes are positioned along a gate-defined wire in a two-dimensional electron gas. Spectroscopy at each probe reveals a hard induced gap and an absence of subgap states at zero magnetic field. However, subgap states emerging at a finite magnetic field are not always correlated between different probes. Moreover, we find that the extracted critical field and effective *g*-factor vary significantly across the length of the wire. Upon studying several such devices, we do however find examples of striking correlations in the local density of states measured at different tunnel probes. We discuss possible sources of variation across devices.

Majorana bound states (MBSs) obey non-Abelian exchange statistics and are potential building blocks of topological qubits.^1,2^ In this context, one-dimensional (1D) semiconductor-superconductor hybrids have been widely studied, where a topological phase transition is accompanied by the emergence of MBSs at the system edges,^3,4^ together with a closing and reopening of the superconducting gap in the hybrid bulk.^5^ Tunnelling spectroscopy provides information about the local density of states (LDOS) and is often used to search for signatures of MBSs.^6^ However, it has been suggested that some of these observations could arise due to trivial reasons such as disorder or inhomogeneity of the chemical potential.^7−15^ Strong local perturbations would effectively segment the wire and, thus, prevent the creation of a global topological phase. It has therefore become clear that a prerequisite for reliably creating MBSs is spatial uniformity of the microscopic properties across the length of the 1D hybrid system. These include the chemical potential, the induced superconducting gap, and the effective *g*-factor.

Information about the bulk density of states of the hybrid region can be inferred by measuring the nonlocal conductance in a three-terminal geometry.^16−20^ However, these measurements are only sensitive to the minimum energy scale of all the bulk states and thus do not immediately reveal local properties. An alternative method to probe the bulk and therefore get information about the wave function of subgap states is to perform local tunneling spectroscopy along the hybrid. While such experiments have been performed in hybrid nanowires, technical difficulties have led to soft superconducting gaps^21^ or additional tunnelling currents that obscure the direct measurement of the LDOS in the hybrid.^22^ Furthermore, the transparency of these tunnel probes is not tunable, thus, preventing a systematic study of LDOS in the bulk. These issues can be mitigated by using a two-dimensional electron gas (2DEG), which offers flexibility in device design and fabrication, allowing one to pattern an arbitrary number of tunable tunnel probes along the 1D hybrid, thus providing information about spatial variation in the LDOS. It has also been proposed that a gate-defined hybrid wire with multiple tunnel junctions is more resilient to inhomogeneous confinement potential, thereby making this device geometry a promising way to probe the LDOS.^23^ Such a geometry has been studied previously in devices based on InAs/Al 2DEGs.^24^ However, the limited number of probes makes it difficult to extract information about the spatial dependence of microscopic parameters along an extended wire.

Here, we study the LDOS of quasi-1D hybrid wires, defined by electrostatic gating in an InSbAs 2DEG with epitaxial aluminum. Several tunnel probes positioned along the wire enable a simultaneous measurement of the position-dependent LDOS. At zero magnetic field, we measure a hard superconducting gap without any subgap states, confirming a strong proximity effect and the presence of clean tunnel junctions. As we increase the magnetic field, we in general do not observe any obvious correlation between the emerging subgap states at neighboring probes, suggesting that these states are localized within 250 nm along the hybrid. Furthermore, we find that the critical field (*B*_c_) and the effective *g*-factor (g*) exhibit significant fluctuations along the wire. In contrast, some devices show remarkably correlated subgap states with spatial extension of more than 1.1 μm. We discuss possible explanations for this inconsistency between different devices.

The InSbAs 2DEG with epitaxial aluminum grown by molecular beam epitaxy has been shown to have a good proximity effect, high g-factor and large spin–orbit coupling.^25,26^ The structures of the multiprobe devices are illustrated in Figure 1, together with the circuit diagram. First a 2.5 μm-long, 130 nm-wide aluminum strip is defined by chemical etching, and nine Ti/Pd normal contacts are deposited along the strip with a center-to-center separation of 250 nm. The aluminum strip remains electrically grounded during measurement, and the bias voltages applied on each contact *V*_i_ (i∈ {1, 2, ··· 9}) can be varied independently. After depositing a 20 nm thick AlOx dielectric layer, a global gate (GG) is deposited. Applying a negative voltage to GG depletes the 2DEG around the Al strip, thereby defining the 1D hybrid wire. At the same time, the 2DEG between any two normal contacts is also depleted, ensuring that no current flows between neighboring tunnel probes. After depositing an additional 20 nm layer of AlOx, nine tunnel gates are deposited over the pinholes in the GG. The applied tunnel gate voltages *V*_Ti_ (i ∈ {1, 2, ..., 9}) control the individual tunnel barrier, allowing one to perform local spectroscopy along the wire. The final image of one of the three measured devices (denoted Device A) is shown in Figure 1c. We also present measurements of two other devices (denoted devices B and C) with the same material but with only four tunnel probes (device images shown in Figure 5).

**Figure 1:**
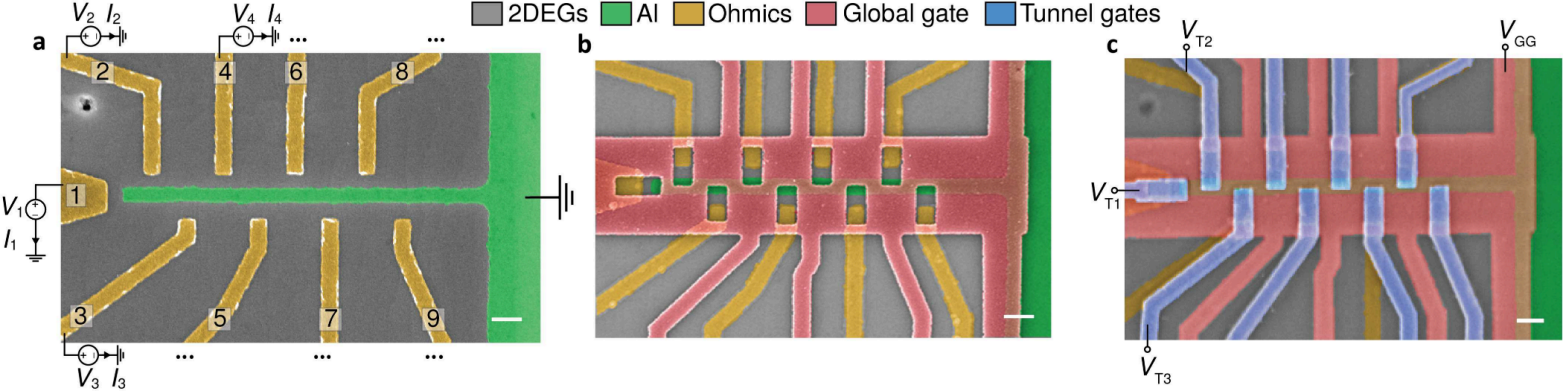
**The multiprobe device.** (a) A false-colored scanning electron microscope (SEM) image of a device with the Al strip and normal contacts. Nine normal contacts are placed from the edge of the wire (“1”) to the bulk (up until “9”). In the circuit diagram, the applied bias voltages and measured currents are shown only for the first four probes for simplicity. (b) SEM image of a device after global gate deposition. (c) SEM image of a device after the tunnel gates deposition. The applied global gate voltages *V*_GG_ and the applied voltages of the first four tunnel gates *V*_T1_ to *V*_T4_ are labeled. The first and second images are from lithographically similar devices. All scale bars are 200 nm.

All measurements were conducted in a dilution refrigerator with a 20 mK base temperature with standard lock-in techniques. More details about the measurement scheme can be found in the measurements methods in the Supporting Information.

We begin the device characterization through tunneling spectroscopy measurements as a function of tunnel gates. Three examples of the measured spectrum are illustrated in Figure 2(a-c). In the tunneling regime (Figure 2d), all three probes show sharp superconducting coherence peaks at approximately ±0.26 meV and a suppression of the in-gap conductance. The tunnel gate voltage *V*_Ti_ affects the transparency of the tunnel junctions. While the out-of-gap conductance varies between around half of the *G*_0_ to nearly zero, the coherence peaks remain at the same energies, as shown in Figure 2a-c. Importantly, we note that there are no obvious charging effects, and no additional subgap states appear over this range of transparency. These spurious states are often observed in hybrid devices and are attributed to a nonuniform confinement potential in the semiconductor junctions.^23,27,28^ The absence of these subgap states at zero magnetic field allows us to extract information about the LDOS in the hybrid wire.

**Figure 2:**
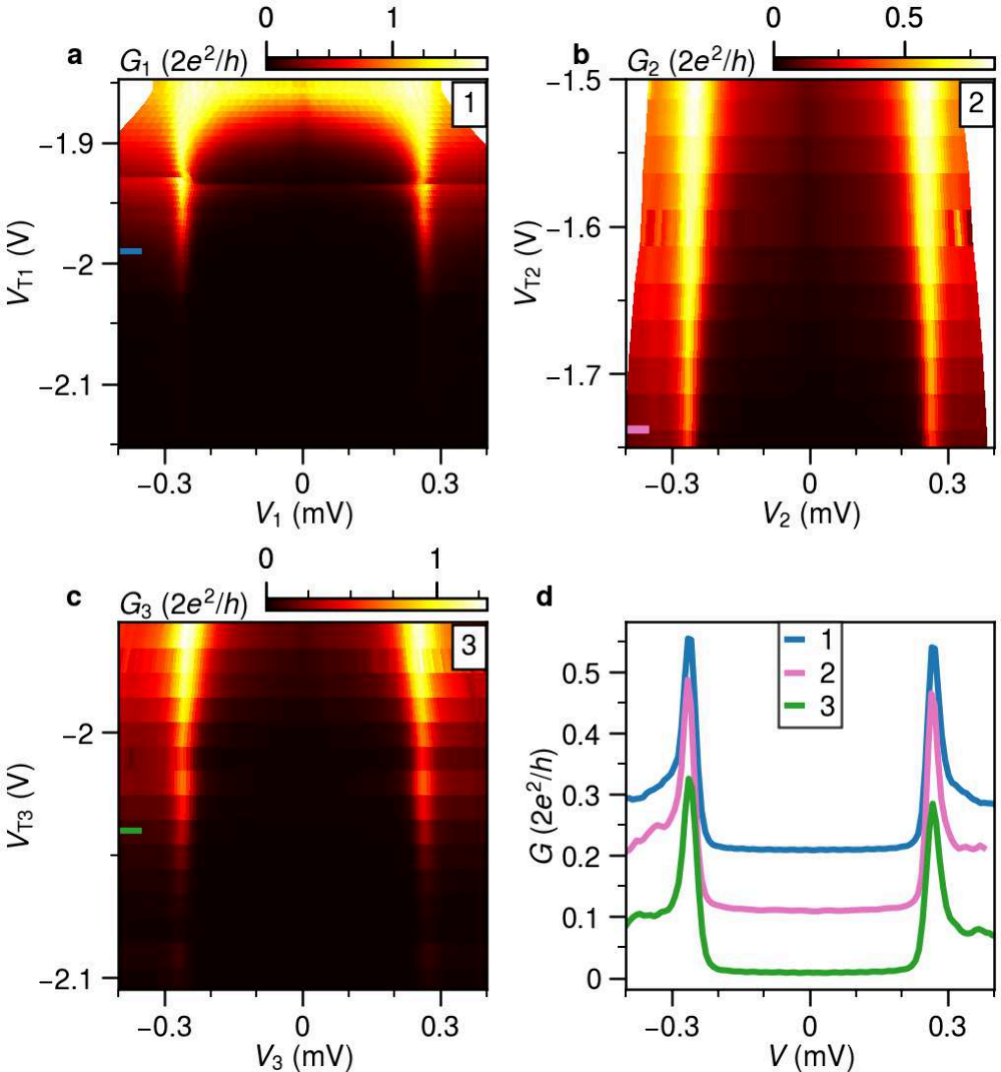
**Hard superconducting gap at zero magnetic field.** (a-c) Tunnelling conductance *G*_i_ as a function of individual tunnel gate TG_i_ and the corresponding applied bias voltage *V*_i_ (i ∈ {1, 2, 3}). With a substantial change of the out-of-gap state conductance, no discrete subgap states are observed within the gap, indicating clean tunnel junctions. Probe numbers are labeled in the top-right corner. (d) Exemplary line traces indicate the presence of two sharp coherence peaks and a hard induced superconducting gap (the lines are laterally offset by 0.1*G*_0_ for clarity). The measured lock-in signals are higher than the noise floor due to the additional parasitic capacitance in the circuit, and a detailed comparison with the numerical derivative of the DC current is made in Figure S1. *V*_GG_ is at −2.6 V.

The precursor of MBSs in a 1D hybrid system is an extended Andreev bound state (ABS) across the entire wire. By applying a large enough magnetic field, a topological phase may arise where the ABS evolves into spatially separated MBSs localized at the ends of the wire. A persisting ZBP is then expected to appear at the edges along with a closing and reopening of the gap in the bulk of the wire. If the spatial separation of a series of tunnel probes is sufficiently small, it should then be possible to map the wave function of the MBSs, which in theory decays exponentially from the wire edge into the bulk.^23^

We measure the tunnelling conductance as a function of the individual applied bias *V*_i_ and a global magnetic field *B* parallel to the aluminum strip, as shown in Figure 3. The tunnel gate voltages are adjusted such that all probes have out-of-gap conductance well below G_0_ and are therefore in the tunnelling regime. The most clear observation from Figure 3 is that there is no systematic correlation in the field evolution of the subgap states moving from the edge to the bulk, indicating the absence of an extended ABS in the wire. For example, the field value where the lowest subgap states cross zero energy differs by about 400 mT between probes 1 and 9. Furthermore, even when we compare the subgap states from neighboring probes (separated by 250 nm), their evolution with a magnetic field seems uncorrelated. For example, the subgap states from probe 2 reach zero energy at around 1.1 T, while this occurs at 1.3 T for probe 3. The measured spectra of probes 3, 5, and 7 look qualitatively similar, but a more detailed comparison shows that the extracted microscopic parameters are different; and thus, these subgap states are also not actually correlated (detailed in Figure 4). The measured tunnel spectra at individual probes can also depend on the chemical potential of the wire. Thus, we also performed the measurements at *V*_GG_ = −1.8 V, just below the threshold voltage required to deplete the bare 2DEG. Similarly uncorrelated subgap states are observed for this set of measurements (Figure S2).

**Figure 3:**
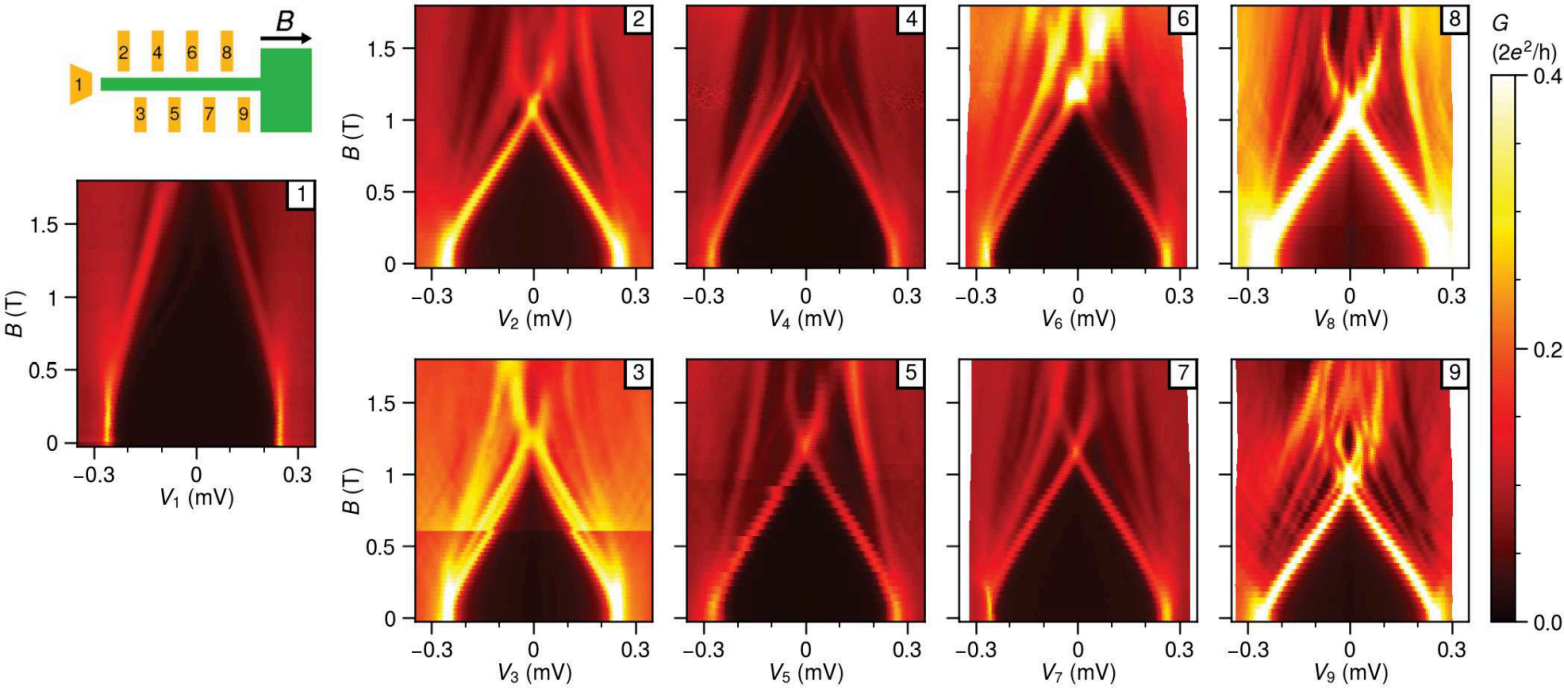
**Field evolution of LDOS for device A.** Tunnelling conductance *G* of each tunnel probe with a schematic of the device. The measurements of probes 1234 and 6789 are obtained by sweeping the four biases at the same time and recording the signals with four lock-in amplifiers. The spectrum of probe 5 is obtained in a three-terminal measurement circuit. No obvious correlation of subgap states between neighboring probes is observed. *V*_GG_ is at −2.6 V.

**Figure 4:**
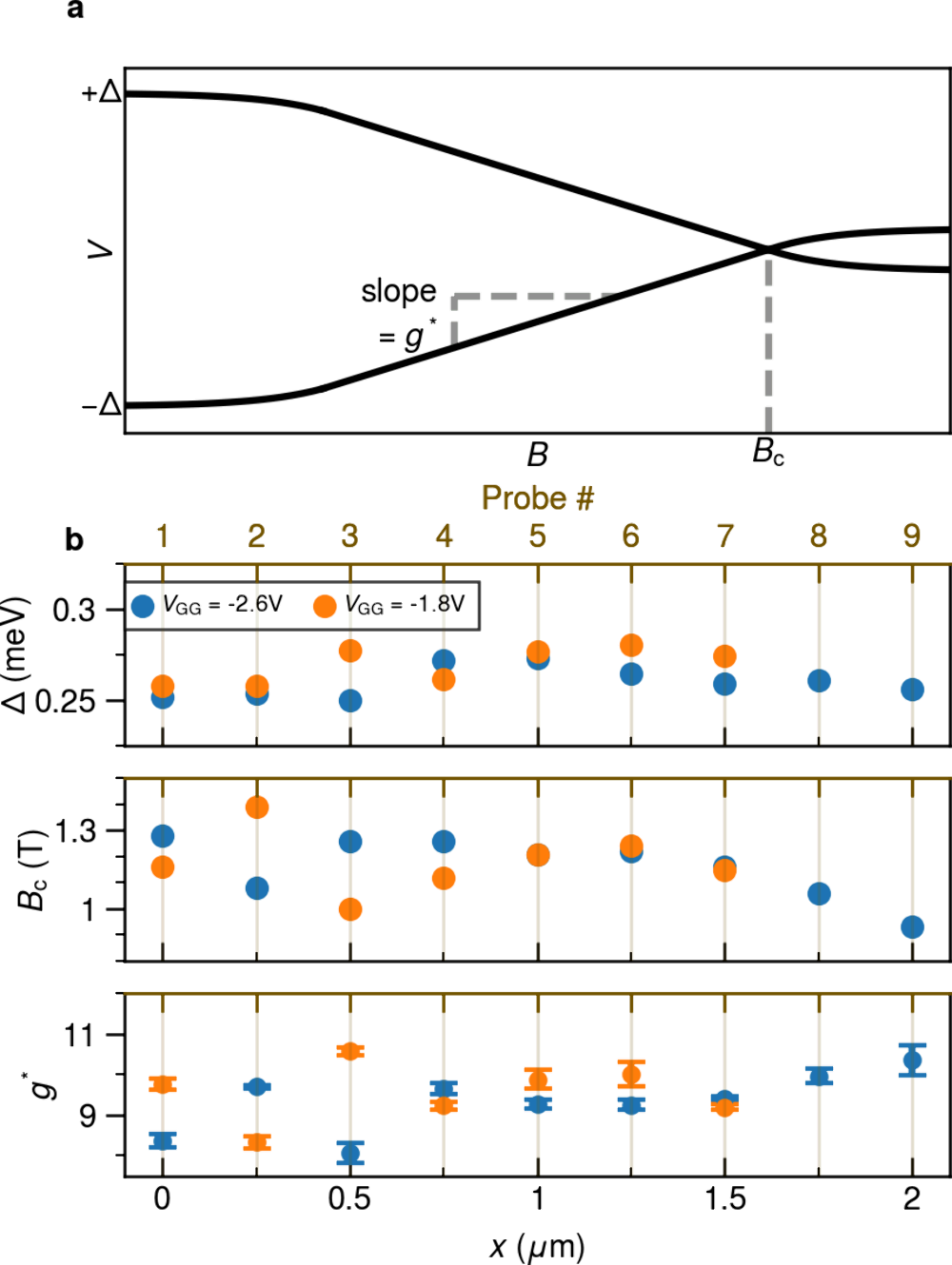
**Spatial dependence of superconducting gap** Δ, **critical field***B*_c_, **and effective g-factor**g*. (a) Sketch of the field dependence of the lowest subgap states. (b) Δ, *B*_c_, and g* are plotted as a function of distance *x* to the edge of the wire (bottom axis) and the corresponding probe number (top axis).

We use the measurement presented in Figure 3 and Figure S2 to extract the spatial dependence of three microscopic parameters in the hybrid: the induced superconducting gap Δ, the critical field *B*_c_, and the effective *g*-factor g* of the lowest-energy subgap states. They are labeled in an exemplar field evolution of the lowest subgap states, as shown in Figure 4a. The size of the induced gap Δ, *B*_c_, and g* characterize the degree of hybridization of the wave function across the superconductor-semiconductor interface. It has been shown that this coupling between the two materials in hybrid nanowires can be modulated by the use of the electric field.^19,29,30^ Δ is determined by locating the applied bias voltages corresponding to the coherence peaks at maxima at *B* = 0. *B*_c_ is defined here as the field value at which the lowest states reach zero energy and is extracted by locating the first local maximum in the zero-bias conductance traces as a function of the magnetic field. g* is defined by ,^31^ where μ_*B*_ is the Bohr magneton, and  is the absolute average of the slope from the linear fitting of the lowest subgap states at positive and negative biases.

As seen in Figure 4b, the induced gap Δ in our devices varies between 0.25 and 0.28 meV along the wire, with an average value of 0.26 mV (GG = −2.6 V) and 0.27 mV (GG = −1.8 V). Additionally, the similar magnitude at two different *V*_GG_ values is probably due to the weak gating effect of the hybrid sections, which is achieved purely by the fringing field of the applied global gate voltages. The spread of the data points can be captured by the calculated coefficient of variation (CV), which is the ratio of the standard deviation to the mean. CV_Δ_ is about 3.1% for GG = −2.6 V and 3.4% for GG = −1.8 V. This variation may be due to mesoscopic variations in the wire or the different tunnel broadening at each probe. For the critical field *B*_c_, however, we find a much stronger variation of the extracted values across different probes. The averaged values are 1.16 T for GG = −2.6 V and 1.18 T for GG = −1.8 V, with the CV reaching about 9.4% in both cases. This significant spread could arise from a nonuniform electrochemical potential in the wire, which is undesirable in realizing a global topological phase transition. The effective *g*-factor is indicative of the extent of hybridization of wave function throughout the cross-sectional interface of the hybrid^29−31^ and eventually determines the required critical field for a topological phase transition. The extracted data here show a large amount of fluctuation, ranging from about 8 to 11. These values are significantly smaller than the *g*-factor of bare InSbAs 2DEG,^25,32^ indicative of hybridization with the superconductor. The error bars originate from the process of linear fitting. For *V*_GG_ = −2.6 V, the mean is 9.4 with a CV of 6.7%, and for *V*_GG_ = 1.8 V, the mean is 9.6 with a CV of 7.5%. The significant spread of *B*_c_ and g*, together with the uncorrelated LDOS shown in Figure 3, indicates a nonuniform chemical potential along the wire, which is nonideal for creating Majoranas.

We repeated similar measurements in two additional multiprobe devices with a similar design. The SEM images of devices B and C are shown in Figure 5a and Figure 5c, respectively. These devices are fabricated with the same 2DEG heterostructures and have the identical shape of the Al strip. However, four normal probes are now arranged with a larger separation of around 500 nm. Basic characterization in Figure S4 confirms a similar hard gap and the absence of subgap states in tunnelling spectroscopy, as the behavior observed in device A. Field dependence measurements are conducted in a comparable tunnelling regime as depicted in Figure 3 for the first three probes from the edge for both devices. Remarkably, the subgap states of probes 1 and 3 now have a remarkably similar dependence on the magnetic field, which we attribute to extended states over 1.1 μm. However, spectroscopy at probe 2 looks different. While some states evolve similarly at all three probes (Figure S6), others do not. This suggests that the wave function of these states is not uniform across the width of the hybrid region. The measurements for device C shows that the lowest subgap states from all probes have the same dependence on the magnetic field, confirming their spatial correlation over 1.1 μm (Figure S6).

**Figure 5:**
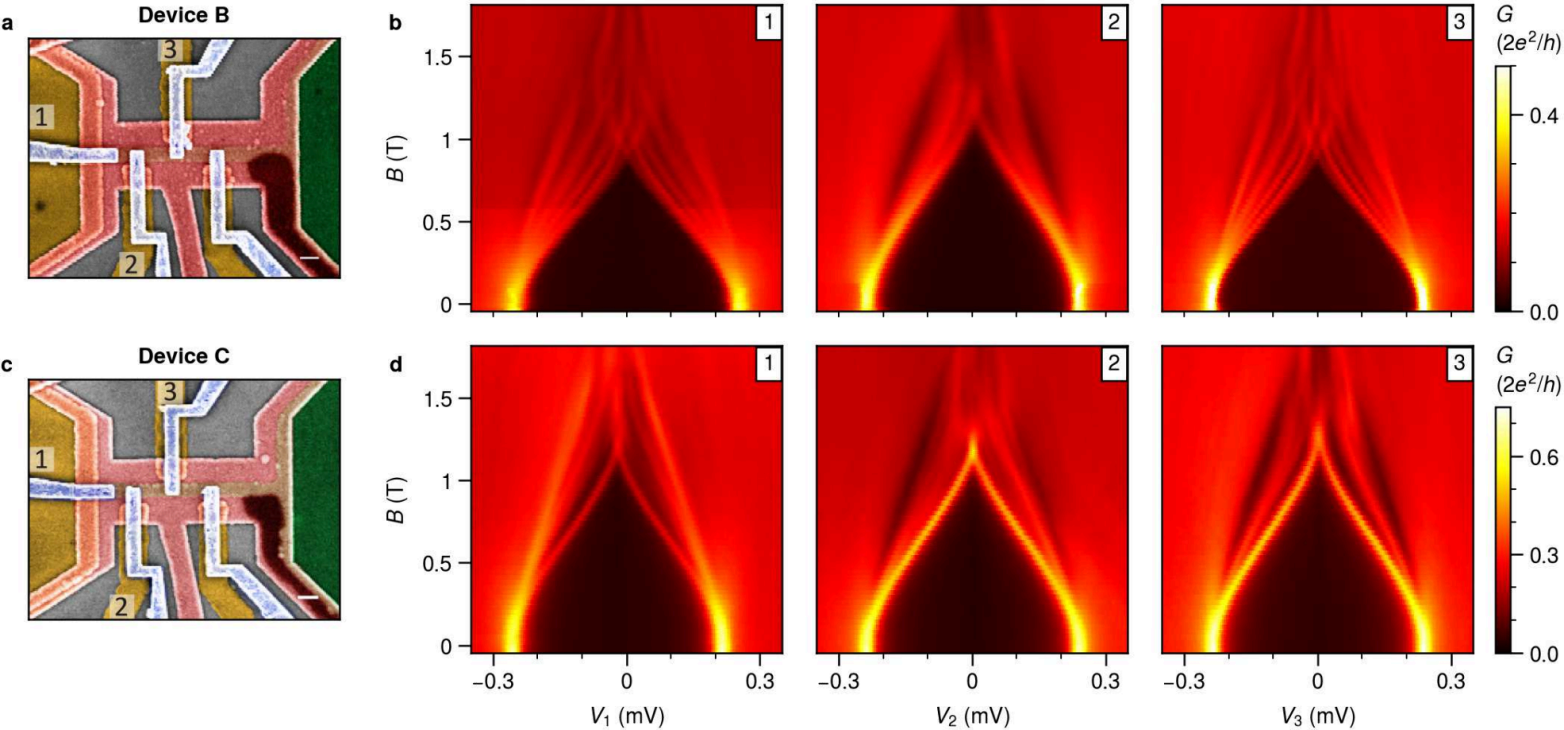
**Field evolution of LDOS on devices B and C**. (a) The false-colored SEM image of device B with a similar shape of the Al strip but only four normal contacts that are separated by around 500 nm. The scale bar here is 200 nm. (b) The field dependence of the leftmost three tunnel probes. The lowest three subgap states spectra have almost the same field dependence between probes 1 and 3, but only the second lowest subgap states are present in probe 2. (c) The false-colored SEM image of another four-probe device C and (d) the field evolution. The lowest subgap states are almost perfectly correlated among all three probes. Details on peak-matching for confirming these correlations are shown in Figure S6.

The variations in the extent of the ABS wave function across different devices warrant a further discussion. We propose a few explanations for this observed discrepancy. First of all, we know that the semiconductor 2DEG used in this study has a typical peak mobility of about 25000 cm^2^/(V s) (which corresponds to a mean-free path of about 250 nm).^25^ Thus, intrinsic disorder could be a factor responsible for the device to device variations. Disorder can also result in a nonuniform chemical potential along the wire, causing the wave function of the ABSs to be spatially nonuniform across the width of the wire. This could partially explain the observations in device B.

Additionally, while the device geometry of device A looks nominally similar to that of device B/C (apart from the number of probes), they actually have different dimensions of the pinholes and dielectric thicknesses (Figure S5), which could potentially lead to different electric fields at the hybrid region. In fact, we observe this experimentally while measuring the tunnelling spectra as a function of tunnel gate voltages at a finite field (Figure S4). The lowest energy subgap states in device A can be affected upon changing the corresponding tunnel gate voltages, in contrast with device B/C, where they remain unaffected. To qualitatively understand this difference, we performed electrostatic simulations in COMSOL, based on the realistic device geometry (Figure S5). We find that in device A, the tunnel gate voltages can create stronger fringing fields in the hybrid region (Figure S3) and thereby effectively lead to the formation of invasive tunnel probes. On the other hand, as a result of the narrower pinholes and the thicker dielectric layers, the tunnel gates in device B/C have a significantly weaker effect on the hybrid region. This is in accordance with the experimental observations whereby devices B/C show stronger correlations between probes as compared to device A. Therefore, it is important to take these electrostatic effects into consideration while designing devices to study the LDOS in hybrid systems.

In conclusion, we have used tunneling spectroscopy to investigate the local density of states in gate-defined wires based on a 2DEG semiconductor-superconductor hybrid structure. This is achieved by implementing a multiprobe device geometry with up to nine side probes placed at different positions along the wire. At zero magnetic field, we observed hard superconducting gaps and clean tunnel junctions, indicating a uniform proximity over 2.5 μm. As the magnetic field increases, subgap states appear and eventually cross zero energy. However, these states are generally not correlated among neighboring probes. The critical field *B*_c_ and effective *g*-factor g* are extracted at two different global gate voltages *V*_GG_ and exhibit significant spatial fluctuations. Measurements from comparable devices show a completely different behavior, where the subgap states evolve identically as a function of magnetic field, suggesting correlations over 1.1 μm. In particular, even in the case of perfect probe-to-probe correlation, we find no clear evidence of a gap reopening, suggesting that the nonuniformity in our devices may be more than what is required to host a global topological phase.^33^

## Data Availability

Raw data and analysis scripts for all presented figures are available at zenodo.org/doi/10.5281/zenodo.11203149.

## Supporting Information Available

Device Fabrication, Measurement methods, COMSOL simulation, Field evolution, Effect of tunnel gates, Identification of subgap states, Characterization of measurement circuits (PDF)
