# Supplementary material for: Spatial Dependence of Local Density of States in Semiconductor-Superconductor Hybrids
Source: Nano Lett. 2024 Oct 18;24(43):13558–63. doi: 10.1021/acs.nanolett.4c03108 (PMC11528430; doi:10.1021/acs.nanolett.4c03108)
Supplement: Supplementary file 1 — nl4c03108_si_001.pdf [file nl4c03108_si_001.pdf]

Supporting Information for: Spatial dependence of local density of states in semiconductor-superconductor hybrids

Qingzhen Wang,[1] Yining Zhang,[1] Saurabh Karwal,[2] and Srijit Goswami[1,†]

[1] *QuTech and Kavli Institute of Nanoscience, Delft University of Technology, Delft, 2600 GA, The Netherlands*
[2] *QuTech and Netherlands Organization for Applied Scientific Research (TNO), Delft, 2628 CK, The Netherlands*

† s.goswami@tudelft.nl

FABRICATION

Three devices were measured to obtain the data presented in the main text and Supplementary (device A, B and C). To prevents the potential intermixing of Al and Sb at high temperature, all the fabrication processes are performed at room temperature unless otherwise specified. We use electron beam lithography to define the required nano-structures and use unexposed PMMA as etch masks. The device fabrication starts by etching the Al and the 2DEG in undesired areas. The Al etch is performed in Transene D etchant at a temperature of 48.2 °C for 9 s. Afterwards, using the same PMMA mask, the 2DEG is etched in a III-V etchant (560 mL deionized water, 5 mL H2O2 and 4 mL 85% H3PO4 and 9.6 g citric acid powder) for 70 s. This leads to an etching depth of about 75 nm and produces a series of un-etched units (called "mesa") which are electrically isolated. Then a sets of fine markers made of Ti/Au are evaporated around each mesa. The purpose of the fine markers is to improve the alignment accuracy between the Al strip, normal contacts and two gate layers to ensure the functionality of the final device. The next step is to define the Al strip, where we carry out a second Al etch in 38.2 °C Transene D for 10 s. Multiple fine normal contacts made of 5 nm/15 nm Ti/Pd are then deposited around the Al strip. Subsequently, a thicker 5 nm/115 nm Ti/Au evaporation defines the contact leads. Afterwards, we deposit a 20 nm-thick global AlOx dielectric at 40 °C. The first layer of gate electrodes (global gate) are formed with a 5 nm/25 nm Ti/Pd evaporation for the fine structures, and then a 5 nm Ti and 115 nm Au evaporation for the coarse gate leads. After depositing the second layer of 20 nm-thick global AlOx dielectric, we deposit a 5 nm/35 nm Ti/Au for the fine structure of the second gate layer and an additional 5 nm Ti/115 nm Ti/Au evaporation for the coarse gate leads.

MEASUREMENT METHODS

For all the measurements, the alignment of the magnetic field with respect to the gate-define wire is expected to be accurate within $\pm$ 10° and calibrated through performing tunnelling spectroscopy of the hybrid section as a function of field angle. During the transport measurement, the aluminium is always electrically grounded. Each available Ohmic lead is biased with both DC and AC voltages and also connected to a current-to-voltage converter, transmitting the outcome to Keithley multimeter DM6500 and lock-in amplifer SR830. Two types of circuits are implemented: for the results presented in Fig. 2, probe 5 in Fig. 3 and Fig. 5b, a common three-terminal circuit as presented in [1] is used. The rest of measurements, in Fig. 3 and Fig. 5d, are acquired with a special multi-terminal circuit. For the case of Fig. 3, the same DC voltage biases are applied on four different probes simultaneously, but each with an AC excitation of distinct frequencies. Thus, we can register four lock-in responses concurrently and perform more efficient measurements. In particular, we have compared the results obtained in the multi-terminal circuit configuration with those obtained with the simplistic two-terminal circuit, and found no qualitative difference in determining the energies of the subgap states Fig. S7.

In the three-terminal circuit, when a DC voltage and a lock-in AC excitation is applied to one Ohmic contact, the another one is kept grounded and vice versa. The amplitudes of AC excitations are always 5 µV and frequencies are 19.99 Hz (lockin-1) and 29.99 Hz (lockin-2). In this way, we can measure the full conductance matrix G in two measurement runs . In the multi-terminal circuits, if two DC biases are swept simultaneously, the frequencies of the AC excitations are 19.99 Hz (lockin-1) and 27.77 Hz (lockin-2). If four DC biases are swept, the frequencies of the AC excitations are 23.33 Hz (lockin-1), 27.77 Hz (lockin-2),13.33 Hz (lockin-3) and 19.99 Hz (lockin-4). Therefore, the tunnelling signals at each tunnel probe can only be demodulated by the single lock-in amplifier whose AC excitation has the same frequency. The voltage-divider effect is accounted for by correcting the real DC voltage drop on each contact with the known fridge line resistances and the resistance from the current-to-voltage converter module. In this way, the evolution of the energies of the subgap states as a function of gate voltage or magnetic field can be correctly resolved. Offsets of the applied voltage biases on each contacts are corrected via averaging the coherence peaks in the conductance line traces.

COMSOL SIMULATIONS

We use AC/DC module in COMSOL Multiphysics 6.1 to simulate the electrostatics in a simplified but realistic geometry. To be more precise, the following specifications are taken directly from the multiprobe devices shown in the main text: the thickness of the 2DEG, the width and thickness of the aluminium strip, the thickness of the AlOx dielectric, the relative position and the size of the pinhole, the thickness of the global gate and tunnel gate layer. Note that the 2DEG is treated as a semiconductor material with a relative permittivity of 17.7. Aluminium is kept

electrically ground and the voltages are applied to the global gate and the tunnel gate. The only variance between device A and device B/C are the different AlOx thickness and the width of the pinhole. The plots shown in Fig. 5 are obtained by performing a parametric sweep of the V_{GG} and V_{TG} with 1 V step.

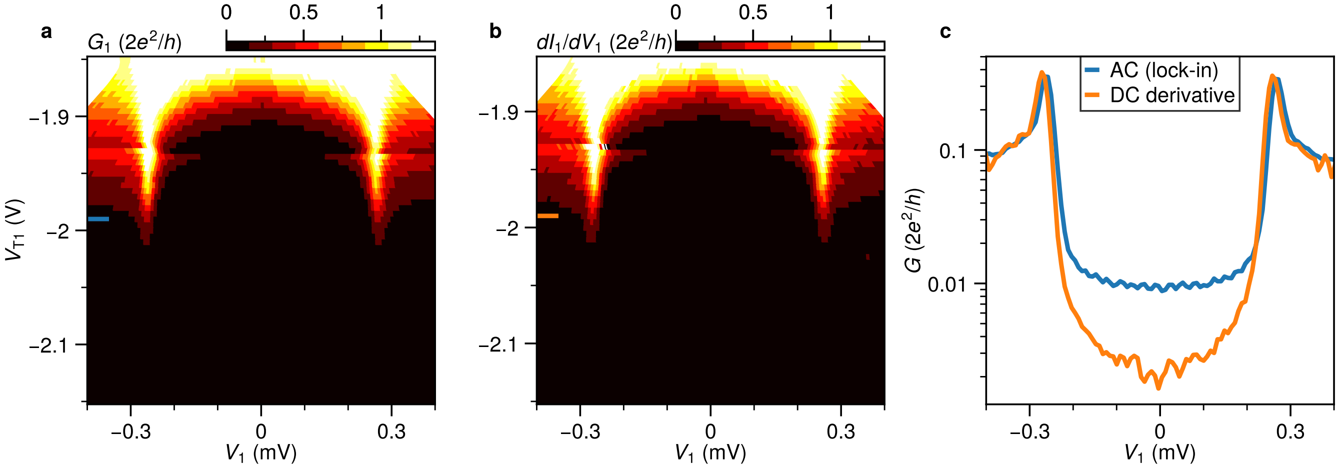

FIG. 1. **Comparison of the AC and DC signals** In this example, the conductance is either measured directly with lock-in amplifier (**a**, Figure 2a in the main text), or calculated with a numerical derivative of the DC current (**b**). The line-cut taken at the same gate voltage (**c**) shows that the in-gap conductance in the DC derivative is about an order of magnitude smaller that of the lock-in signal. This is because the additional parasitic components in the circuit induce an additional phase shift, thus making the amplitude of the measured lock-in signal higher. A Savitzky–Golay filter of window length of 5 is applied for the DC current before taking the numerical derivation.

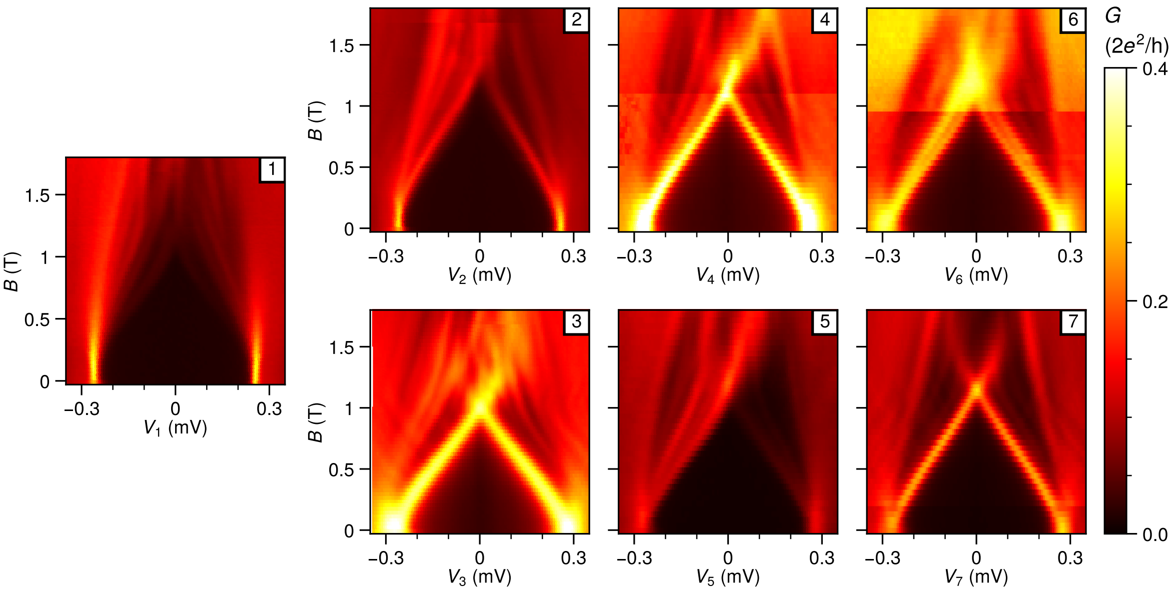

FIG. 2. **Field evolution of LDOS at $V_{\mathrm{GG}} = -1.8\,\mathrm{V}$: Device A.** Similar to the behavior observed in main text FIG. 3, no obvious correlation of subgap states between neighboring probes are observed. Note that the field evolution has only been recorded for the first seven probes because of difficulties in adjusting the the tunnel gates for Probe 8 and Probe 9.

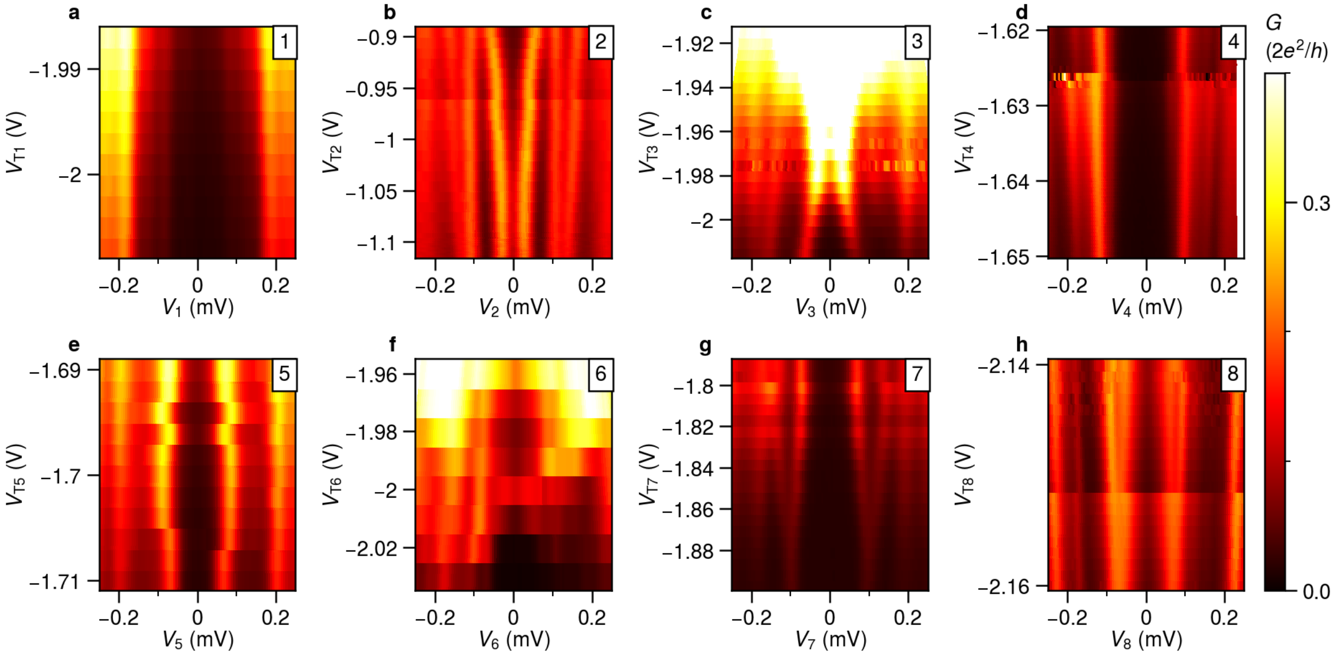

FIG. 3. **Effect of tunnel gates on spectroscopy: Device A**. The probe numbers are indicated in the top left of each figure. Overall, the subgap states do not stay at constant energies upon changing the corresponding tunnel gate voltages, indicating that the tunnel gates can have an effect on the hybrid region. This observation also indicates that the electrostatics can lead to false-positive observation of correlated subgap states, as a result of fine-tuned gate voltages.

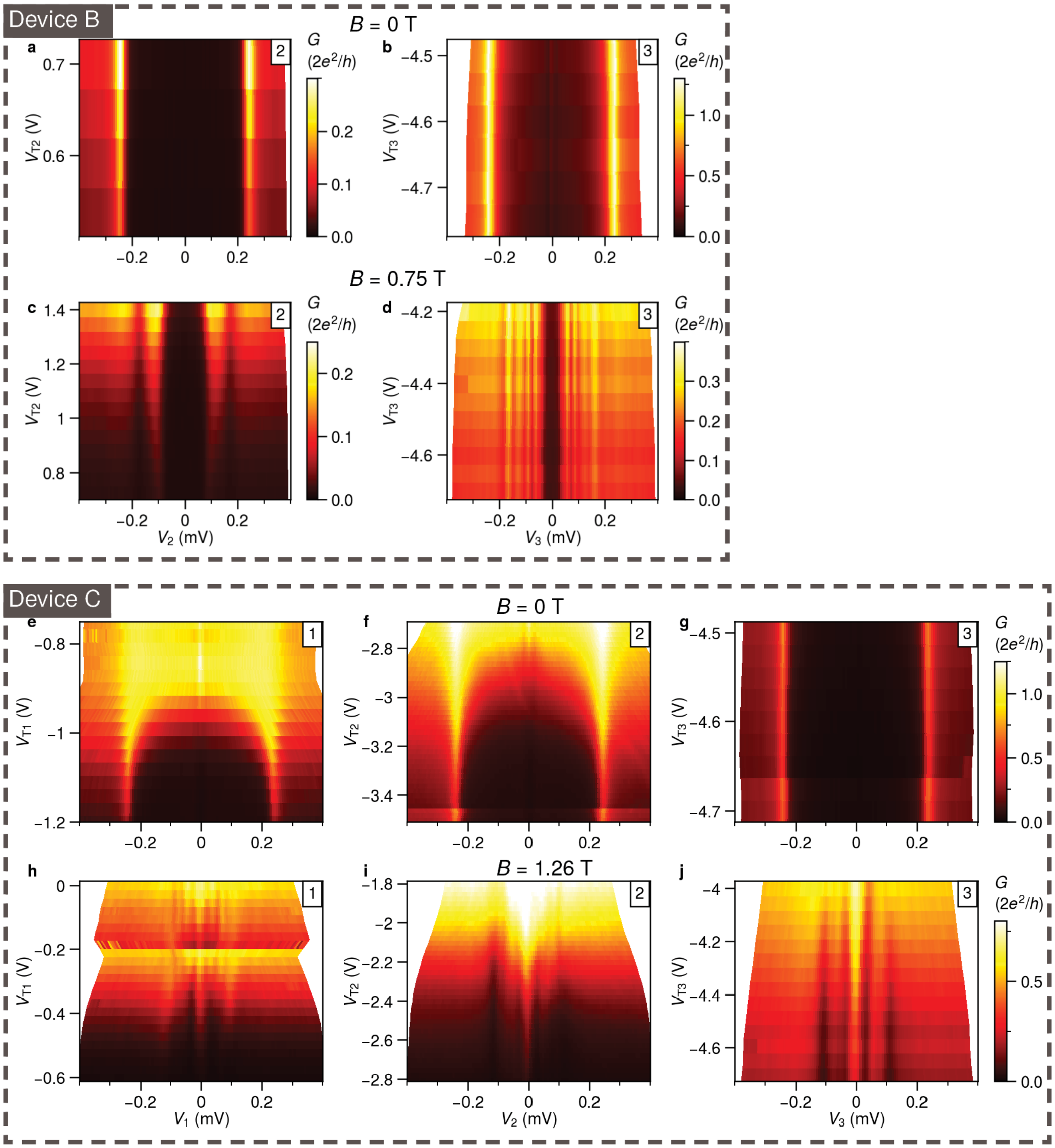

FIG. 4. **Effect of tunnel gates on spectroscopy: Device B and C.** Hard gaps without the subgap states (**(a-b) and (e-g)**) are observed are observed for both devices. At a finite field, the subgap states stay at almost the same energies as a function of tunnel gate voltages over a large range of transparency (**(c-d) and (h-j)**). Note that for device B, the tunnel gate T1 has almost no tunability of the junction transparency, and therefore only the T2 and T3-dependence are shown here.

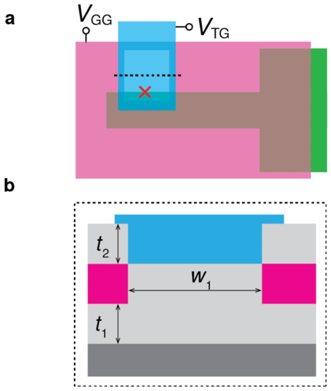

	Device A	Device B/C
t_1	20 nm	30 nm
t_2	20 nm	30 nm
w_1	150 nm	90 nm

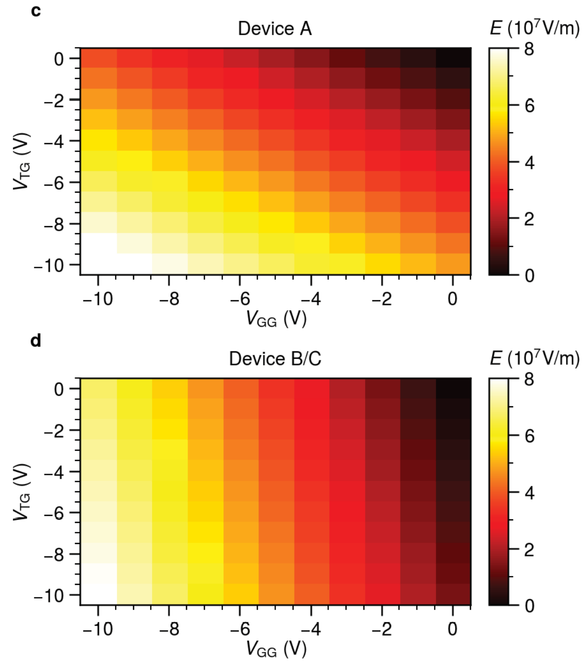

FIG. 5. **COMSOL simulations of devices.** **(a)** Top view of the mulitprobe device, with only one representative pinhole on the global gate and only one tunnel gate. The simulated electric field strength at the red cross is chosen to represent the effect of the applied global gate voltages V_{GG} and tunnel gate voltages V_{TG}. **(b)** A cross-sectional view of the device stack, taken along the dashed line in **(a)**. The length scales that are relevant for the electrostatics are the thickness of the first ALD layer (t_1), the thickness of the second ALD layer (t_2) and the width of the pinhole (w_1). The table summarizes these parameters. **(c)** The simulated electric field norm for device A as a function of V_{GG} and V_{TG}. The change in the field amplitude as a function of only TG or GG of the same range is very similar. **(d)**The simulated electric field norm for device B/C. In contrast to **(c)**, now the global gate has a much stronger effect on the 2DEG than the tunnel gate. This is consistent with the overall thicker dielectric thickness ($t_1 + t_2$) and the smaller pinhole width (w_1) in device B/C, compared to device A.This may explain the weaker tunnel gate effect on the measured spectrum Fig. 4, and the relatively stronger gating effect in device A Fig. 3. More details about the COMSOL simulation can be found in the methods section.

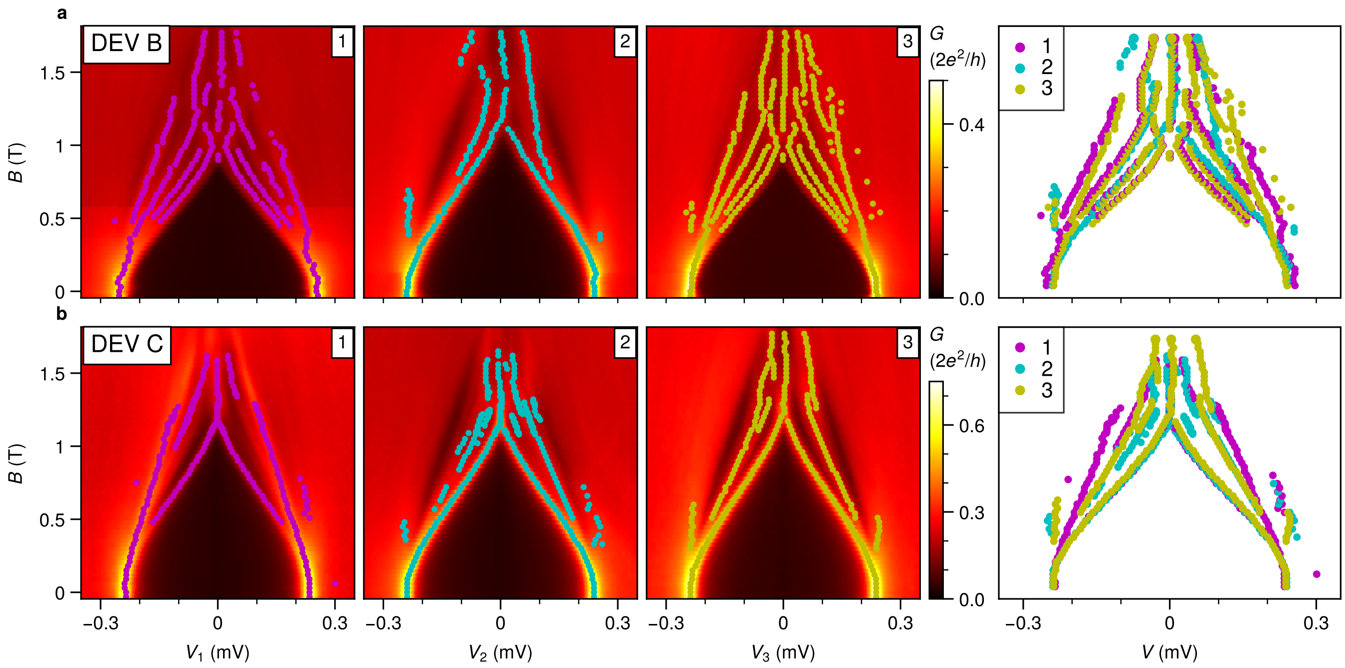

FIG. 6. **Identification of subgap states in Figure. 5: Device B and C**. At each field value, we find the local maxima in the tunnelling conductance and overlay these states from different probes in the last plot of each row. For device B ((**a**),top row), the lowest subgap states of probe 1 and probe 3 show identical dependence on magnetic field, but are absent in the spectra in probe 2. However, the evolution of the second-lowest states of probe 1 and 3 matches well with that of the lowest states of probe 2. For device C((**b**),bottom row), the lowest energy subgap states from the three probes show identical dependence.

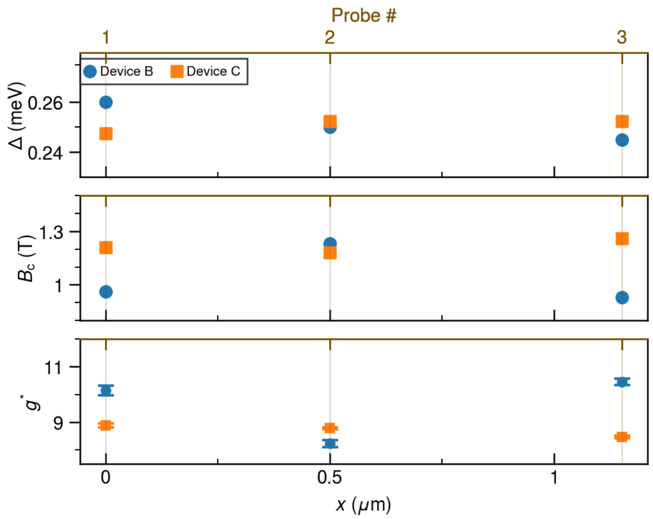

FIG. 7. **Spatial dependence of superconducting gap Δ, critical field B_c and effective g-factor g^* of the lowest subgap states: Device B and C.** For device B, the value of B_c and g^* at probe 2 is clearly different from those at probe 1 and 3. For Device C, however, these parameters are nearly identical at various probes.

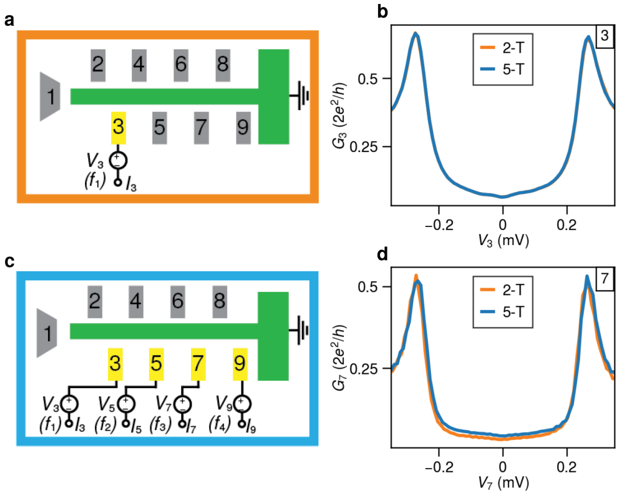

FIG. 8. **Comparison of the measured signal with different circuit configurations.** Measurements here are performed either with a two-terminal circuit diagram (with the voltage bias applied to either the probe 3, as shown in **(a)** or the probe 7) or a five-terminal circuit (with voltage biases applied simultaneously to four probes, shown in **(c)**). The measured conductance via two kinds of circuits are then plotted together for probe 3 (**b**) and probe 7 (**d**). There is no significant difference regarding the positions and heights of the coherence peaks between these two measurements, confirming the validity of the five-terminal circuit configuration for investigating the subgap states.

REFERENCES

[1] Esteban A. Martinez; Andreas Pöschl; Esben Bork Hansen; May An Y. van de Poll; Saulius Vaitiekėnas; Andrew P. Higginbotham; and Lucas Casparis. Measurement circuit effects in three-terminal electrical transport measurements. *arXiv:2104.02671*, 2021-04-06. Available at: https://arxiv.org/abs/2104.02671.
